# Olfactory testing in infants with perinatal asphyxia: Enhancing encephalopathy risk stratification for future health outcomes

**DOI:** 10.1016/j.neubiorev.2025.106029

**Published:** 2025-01-26

**Authors:** Serafina Perrone, Virginia Beretta, Maria Luisa Tataranno, Sidhartha Tan, Zhongjie Shi, Elena Scarpa, Valentina Dell’Orto, Sebastiano Ravenda, Chiara Petrolini, Maria Maddalena Brambilla, Paola Palanza, Eloisa Gitto, Francesco Nonnis-Marzano

**Affiliations:** a Neonatology Unit, Department of Medicine and Surgery, University of Parma, Pietro Barilla Children’s Hospital, Parma 43121, Italy; b Department of Neonatology, Wilhelmina Children’s Hospital, University Medical Centre Utrecht, Utrecht 3584 EA, the Netherlands; c Department of Pediatrics, Wayne State University School of Medicine, Detroit, MI, USA; d Stress Physiology Lab, Department of Chemistry, Life Sciences and Environmental Sustainability, University of Parma, Viale delle Scienze 11, Parma 43125, Italy; e Unit of Behavioral Biology, Department of Neuroscience, University of Parma, Viale delle Scienze 11/A, Parma 43125, Italy; f Neonatal and Pediatric Intensive Care Unit, Department of Human Pathology of the Adult and Developmental Age "Gaetano Barresi", University of Messina, Messina 98125, Italy; g Department of Chemistry, Life Sciences and Environmental Sustainability, University of Parma, Viale delle Scienze 11, Parma 43125, Italy

**Keywords:** Perinatal asphyxia, Newborn, Olfactory system, Risk stratification, Biomarkers

## Abstract

Perinatal asphyxia (PA) is a leading cause of neonatal morbidity and mortality, often resulting in long-term neurodevelopmental challenges. Despite advancements in perinatal care, predicting long-term outcomes remains difficult. Early diagnosis is essential for timely interventions to reduce brain injury, with tools such as Magnetic Resonance Imaging, brain ultrasound, and emerging biomarkers playing a possible key role. Olfaction, one of the earliest senses to develop, may provide valuable insights into long-term neurodevelopmental outcomes following PA due to its intricate neural connections with regions responsible for memory, emotion, and homeostasis. Newborns demonstrate early olfactory abilities, such as recognizing maternal odors, which are vital for bonding, feeding, and emotional regulation. These responses are processed by a network of brain regions, including the olfactory bulb (OB), piriform cortex, amygdala, and orbitofrontal cortex. Hypoxic injury to these regions, particularly the OB, may disrupt olfactory processing in infants with PA, potentially affecting their cognitive and social development. Investigating the relationship between olfactory system development and perinatal brain injury could lead to innovative diagnostic and therapeutic approaches. Further research, including clinical and animal studies, is necessary to fully explore the potential of olfactory assessments in predicting outcomes after PA. This educational review explores and discusses the potential of olfaction as a predictor of long-term outcomes and a tool for risk stratification following PA, opening new pathways for interventions and improved care.

## 1. Introduction

Perinatal asphyxia (PA), caused by reduced blood flow or oxygen levels around the time of birth, remains a significant contributor to neonatal mortality and long-term neurodevelopmental disabilities. The incidence of sever PA is estimated at 2 per 1000 live births in developed countries but is tenfold higher in developing regions due to limited access to maternal and neonatal care (Gebregziabher et al., 2020; Dubie et al., 2021). While advancements in perinatal medicine have improved survival rates, the neurodevelopmental outcomes after hypoxic-ischemic encephalopathy (HIE) remain concerning, with 20 % mortality and 25 % of survivors experiencing long-term neurological deficits, including cerebral palsy and cognitive impairments (Lee and Glass, 2021; Mota-Rojas et al., 2022; Cornet et al., 2023).

Early identification of brain injury is critical for timely intervention and improving outcomes, particularly for infants with mild-to-moderate HIE whose prognoses remain uncertain (Bouiller et al., 2016; Nguefack et al., 2022).

Current diagnostic tools, including MRI, neurological examinations, and amplitude-integrated electroencephalography (aEEG), are highly effective for severe cases but less reliable for predicting long-term outcomes in milder forms of HIE (Del Río et al., 2016; Mitra et al., 2019). Furthermore, these tools are resource-intensive and often unavailable in low-resource settings, leaving many infants outside of standard follow-up programs. Moreover, in most centers, these mild-moderate cases of HIE will not receive a brain MRI of continuous brain monitoring and will not enter the standard follow up programs.

To personalize therapeutic approaches and enhance prediction of long-term outcomes, it is vital to better stratify the risk of long-term neurodevelopmental impairments following hypoxic-ischemic insults of any grade, by evaluating emerging biomarkers (Shi et al., 2022a). A growing body of research has focused on biochemical biomarkers for predicting neuronal injury and long-term neurodevelopmental outcomes. Biomarkers of oxidative stress (e.g., free iron, F2-isoprostanes, and advanced oxidative protein products), lactate levels, and brain-specific proteins such as protein S100, neuron-specific enolase, and glial fibrillary acidic protein have shown promise (Negro et al., 2018; Goswami et al., 2020). Despite their potential, these biomarkers face challenges in clinical application, including limited neonatal-specific reference data, sensitivity and specificity issues, and variability in biological response. Furthermore, biomarkers must cross the blood-brain barrier and persist in detectable concentrations, conditions that are not consistently met in neonates. As a result, while these tools may complement traditional diagnostics, they have yet to achieve widespread clinical adoption.

Emerging research highlights the potential of olfactory testing as a non-invasive and cost-effective tool to complement existing methods. The olfactory system, among the earliest sensory systems to develop and mature, is particularly vulnerable to hypoxic-ischemic injury due to its proximity to brain structures and high metabolic demands. Animal studies demonstrate significant damage to olfactory structures following perinatal hypoxia-ischemia (HI), with abnormal motor responses to odors observed in models of cerebral palsy (Drobyshevsky et al., 2006, 2012; Dubrovskaya et al., 2022; Shi et al., 2022b). These findings suggest that olfactory dysfunction could serve as an early indicator of brain injury and provide critical insights into long-term outcomes. Integrating olfactory assessments into neonatal care could offer a novel approach to risk stratification for infants with HIE.

This review explores the possible potential role of olfactory testing as a predictor of long-term outcomes in infants with HIE following PA. By combining olfactory assessments with biochemical, imaging, and electrophysiological approaches, we propose a multimodal strategy for early detection of brain injury, personalized therapeutic interventions, and enhanced neurodevelopmental outcomes.

Despite seeming very promising, the current review is mostly exploratory since only a few studies have documented the damage to the structures involved in olfactory perception following perinatal HI in neonatal animal models.

## 2. Pre-clinical models of HIE and the olfactory response

Dobryshevsky and colleagues investigated olfactory epithelium injury, following fetal HI in rabbits (Drobyshevsky et al., 2006). They used Manganese-Enhanced MRI, a non-invasive technique that measures neuronal functional activity based on the neurons’ ability to uptake manganese ions through calcium channels and actively transport them via their axons, and found that in the HI group, there was a notable reduction in the signal from manganese uptake by the neurons of the olfactory bulb, alongside a reduction in olfactory epithelium thickness and a decrease in the number of mature olfactory neurons compared to controls. In utero HI resulted in impaired postnatal response to odorants and poor feeding.

As nitric oxide (NO) plays a key role in the development and functioning of the olfactory system (Bredt and Snyder, 1994; Wilson et al., 2007), mediating formation of synaptic connections in developing and regenerating olfactory receptor neurons (Roskams et al., 1994), Dobryshevsky and colleagues, manipulated NO levels in the olfactory neuron in postnatal kits by administration of intranasal NO donors or a highly selective nNOS inhibitor (Drobyshevsky et al., 2012). They found that NO Synthase is present in rabbit olfactory epithelium during the first three postnatal weeks. Modulation of olfactory transduction by NO levels following perinatal HI may affect early functional development of olfaction by decreasing NO-dependence of olfactory signal transduction.

More recently (Dubrovskaya et al., 2022), demonstrated that prenatal hypoxia during the period of active formation of cortical brain structures in rats significantly impairs the processes underlying the development and biochemical characteristics of the central parts of the olfactory system, as the entorhinal cortex and hippocampus. During the postnatal development of rats, the authors observed a reduction in the effectiveness of olfactory function, apparent as lower levels of food search productivity, with impairment to recognition of the odors in rats subjected to antenatal hypoxia. They also utilized microphotographs of the piriform cortex (PC) and found clear signs of neuronal destruction, including edematous processes and swollen neurons in the hypoxia-ischemia group relative to the control group. They also measured the percentage of different cell populations in the area of the rat PC after the insult, reporting significant decreases in non-pyramidal cells and pyramidal neurons, with an increase in the percentage of glial cells (Dubrovskaya et al., 2022).

Amakhin et al. (2022) investigated long term effects of prenatal hypoxia in 3-week-old rats. Antenatal hypoxia increased the excitability of neurons in the entorhinal cortex and hippocampus of rat offspring. Through immunochemistry it was found a reduction in the total number of active neurons in the entorhinal cortex. Structural and biophysical changes in the neuronal circuitries may lead to hyperexcitability in limbic structures and lower seizure thresholds in the hypoxic group.

Moreover, it was demonstrated that prenatal hypoxia determines significant decrease of neuropeptidase neprilysin and synaptophysin in cerebral cortex and hippocampus. Administration of Caspase-3 Inhibitor following prenatal hypoxia restores neuropeptidase neprilysin expression, the number of dendritic spines in the entorhinal and parietal cortices, hippocampus and rescues rat olfactory function in food search and odor preference tests (Vasilev et al., 2021). Neuropeptidase neprilysin participates in olfactory signaling, involving somatostatin and l Aβ peptides levels, whose receptors are widely distributed in entorhinal, parietal cortex and hippocampus (Nocera et al., 2019).

The potential influence of odor was investigated in a study by Shi and colleagues, who developed a novel scoring system in rabbits to assess cognitive function during the neonatal period following prenatal HI. The authors implemented a conditioning protocol in which kits from each litter were randomly assigned. In this protocol, a consistent human feeder wearing a laboratory coat provided care to the rabbit kits for nine days prior to cognitive testing. They compared normally developing kits born after uterine ischemia with naïve kits. The study revealed that conditioned kits, both naïve and HI-exposed, demonstrated a significant preference for the face of the feeder but not for the laboratory coat. The authors suggested that rabbits might still detect subtle odor differences between the feeder and a bystander (Shi et al., 2024).

These findings underscore the extensive damage caused by perinatal hypoxia-ischemia across multiple levels of the olfactory system, from the sensory neurons in the olfactory epithelium to the cortical areas responsible for processing odors, emphasizing the importance of monitoring olfactory function in the context of neurodevelopmental outcomes following PA (Table 1).

### 2.1. The sense of smell in humans

Olfaction is one of the most ancient and essential human senses. On the other hand, from an evolutionary point of view survival in vertebrates is strongly connected to the application of olfaction in different life strategies, from feeding to social development (Rilling and Young, 2014). The ability to smell is crucial from birth (Logan et al., 2012), so the fundamental features of Olfactory Bulb (OB) circuitry must be present and functional from the earliest postnatal ages. The anatomical formation of the olfactory system begins around the 4th week of gestation, with functionality emerging around the 8th week (Lipchock et al., 2011; Sarnat et al., 2017). From the 29th week of gestation, MRI scans can reliably detect the OB, and their hypoplasia or aplasia are identifiable in both prenatal and postnatal imaging (Azoulay et al., 2006).

At birth, the main structures of the olfactory system—including the OB, olfactory tubercle, entorhinal cortex, and PC—are present and functional in healthy newborns. However, the olfactory system continues to mature through processes like neuronal differentiation, synaptogenesis, and myelination during postnatal development (Sarnat and Yu, 2016; Tufo et al., 2022). This ongoing plasticity enables the refinement of olfactory processing and the development of more complex functions in the early years of life. Notably, neuroblast proliferation from neural stem cells has been observed in the postnatal brains of mammals, including humans, contributing to brain development and adaptability. Recent studies by Akter et al. (2021) have shed light on postnatal neurogenesis and neuronal migration in rodents and primates, emphasizing the rostral migratory stream toward the olfactory bulb and its connection to the neocortex in humans, which persists for several months after birth. The migration patterns and final roles of neuroblasts appear to vary based on their environment, destination, and functions within the brain (Akter et al., 2021). The olfactory system detects and processes airborne volatile chemical stimuli, known as odorants, providing critical information about food, our bodies, other individuals, animals, plants, and the surrounding environment (Touhara and Vosshall., 2009; Sharma et al., 2019).

**Fig. 1.**
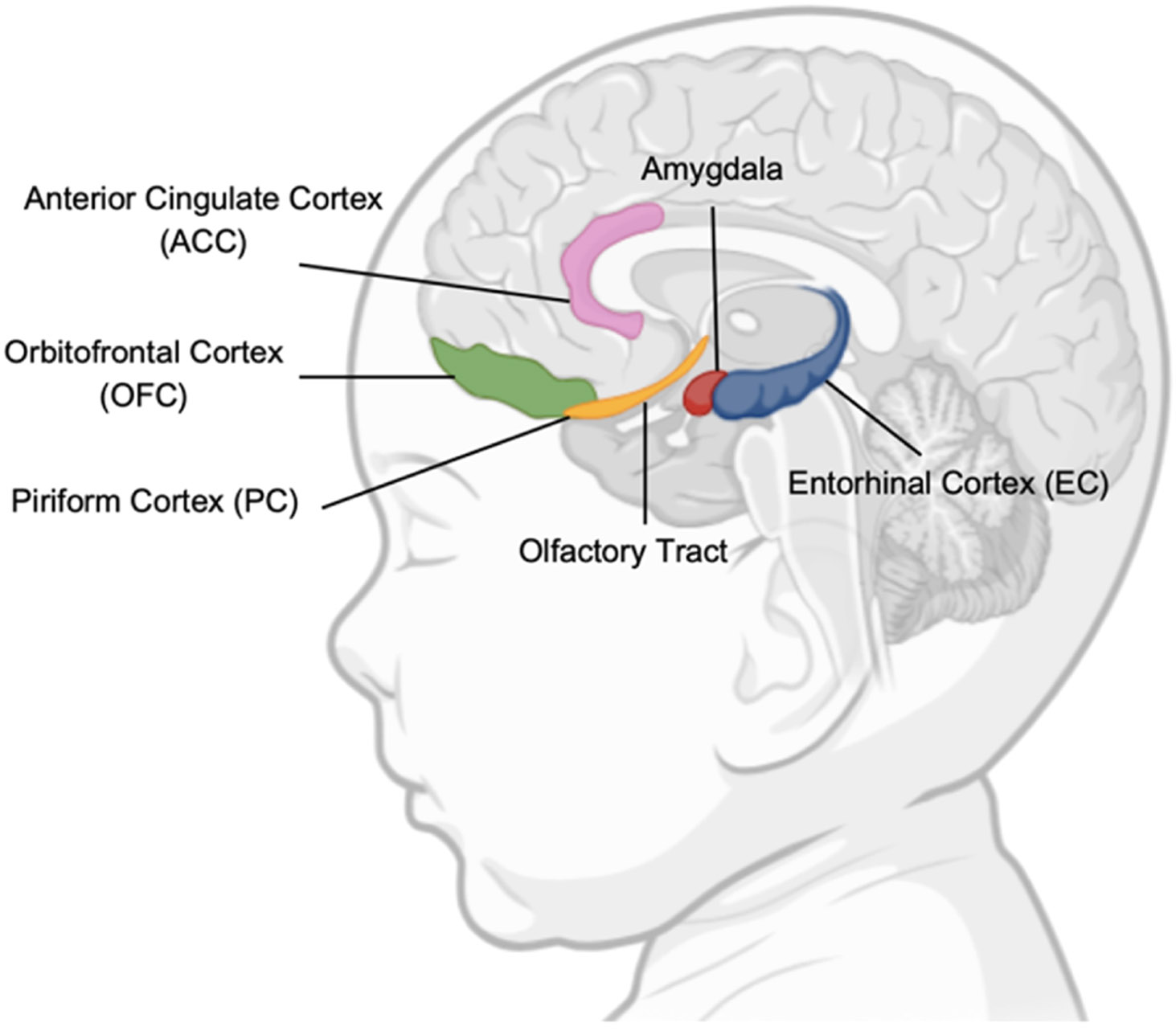
Illustrates key brain areas in neonatal olfactory processing. Piriform Cortex (PC): Processes sensory input for odor recognition; Entorhinal Cortex (EC): Links smells to early memory; Orbitofrontal Cortex (OFC): Supports integration of olfactory and other sensory inputs; Anterior Cingulate Cortex (ACC): Connects olfactory and sensory attention, including pain processing.

Fig. 1 shows this complex network of regions, which highlights the profound importance of the olfactory system, not only in detecting odors but also in its integration with emotional, memory, and homeostatic systems, making it a critical sensory modality in early life and a window into brain development and function (Fig. 1).

**Fig. 2.**
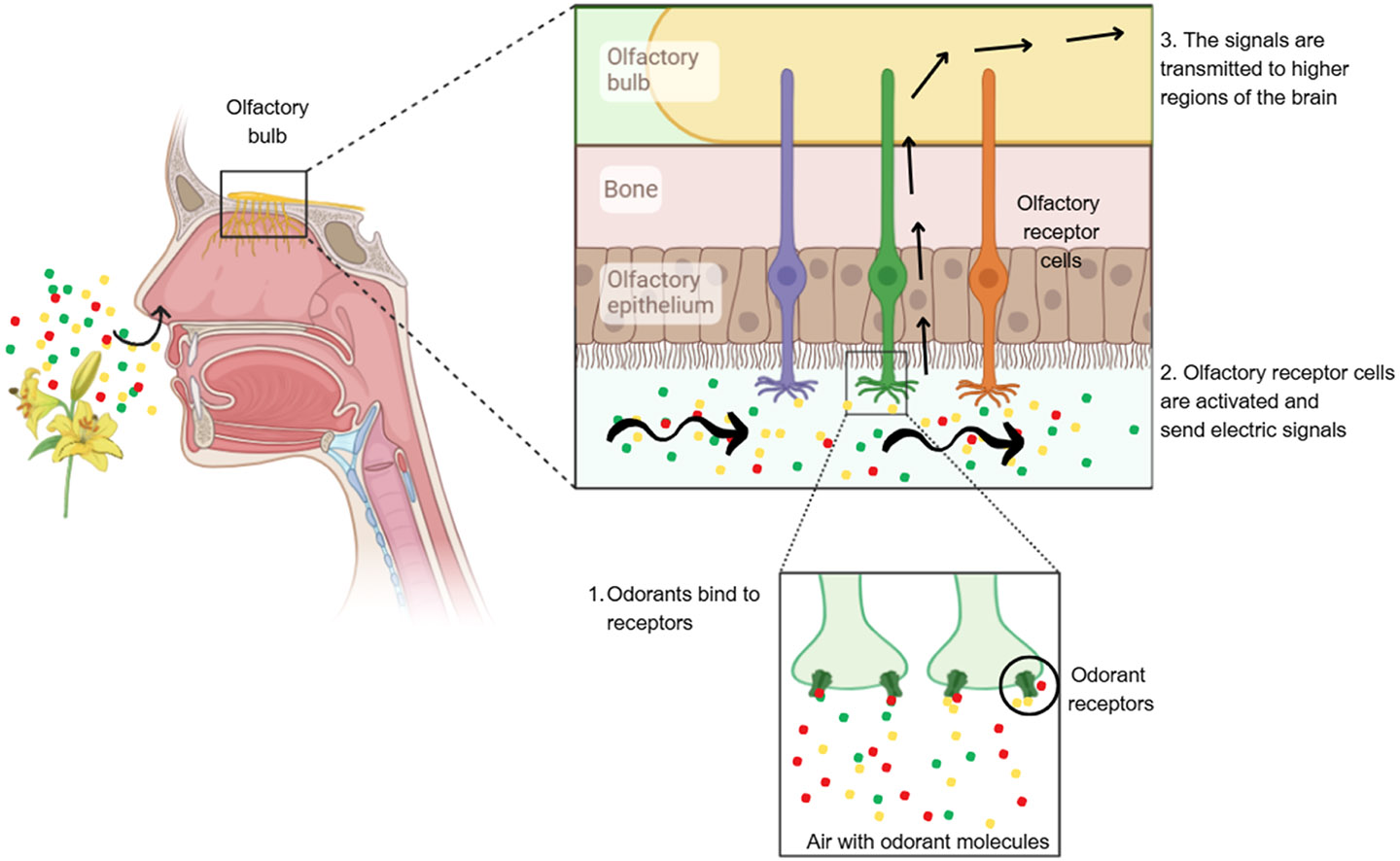
Schematic representation of the olfactory system. Odorant molecules enter the nasal cavity and bind to specific receptors located on the olfactory receptor cells within the olfactory epithelium (1). This binding activates the receptor cells, generating electrical signals (2). The signals are then transmitted to the olfactory bulb and further relayed to higher brain regions for processing and perception of smell (3).

The olfactory sensory neurons (OSNs) located in the olfactory epithelium of the nasal cavity. These specialized neurons are equipped with olfactory receptors that transduce chemical signals from molecules into electrical impulses, initiating the sensory cascade (Fig. 2). Once these impulses are generated, they trigger the release of neurotransmitters in the olfactory bulb, the first relay station in the olfactory pathway. The homeostasis and structural integrity of the olfactory epithelium is maintained by a combination of mucus secreted by Bowman’s glands and the supportive actions of sustentacular cells. Bowman’s glands produce mucus that traps and dissolves odor molecules, facilitating their interaction with olfactory receptors, while sustentacular cells provide physical support, metabolic exchange, and detoxification within the olfactory epithelium (Branigan and Tadi, 2024).

Signals from the olfactory receptors are carried by the olfactory nerve to the OB, where they are processed and relayed via the olfactory tract to various cortical regions. These regions include the olfactory tubercle, PC, amygdala, and entorhinal cortex, each playing a distinct role in odor perception and processing. Together, they enable the identification of odors, the association of smells with emotions, and the connection of scents to memories (Purves et al., 2001). The amygdala plays a critical role in the emotional responses to smells, particularly those associated with danger or pleasure, while the entorhinal cortex links odors to episodic memory. Additionally, olfactory information is integrated with signals from other cortical regions, including the orbitofrontal cortex (OFC), hippocampus, anterior cingulate cortex (ACC), entorhinal cortex, and hypothalamus further refining our perception of smells and their behavioral significance (Smith and Bhatnagar., 2019). The OFC is critical for the conscious perception of odors and decision-making related to smell, while the thalamus acts as a relay center, processing sensory input. The entorhinal cortex is involved in memory processing, particularly in the formation of associations between sensory experiences and spatial or temporal contexts, and the hypothalamus, the body’s homeostasis regulator, connects smells to physiological states, such as hunger or sexual arousal (Aqrabawi and Kim, 2018; Zhao et al., 2023). These regions are often impaired in infants with HIE, which can disrupt normal olfactory processing and potentially impact cognitive and emotional development (Kebaya et al., 2023).

### 2.2. Cortical olfactory processing in newborns

In newborns, as in adults, the first neuronal level of olfactory information processing occurs in the OB, by receiving information from the olfactory epithelium situated in the nasal cavity. However, a critical difference in neonates is the absence of fully developed air-filled sinuses, which in adults introduce artifacts in neuroimaging techniques such as functional MRI due to the air-tissue interfaces (Wilson and Sullivan, 2011; Ekanayake et al., 2023). This lack of sinus development facilitates clearer detection of olfactory bulb activation in neonatal imaging studies. The absence of sinus-related distortions in newborns allows for a more straightforward capture of olfactory processing, leading to insights into how the olfactory system develops and functions in the early stages of life. From the OB, olfactory information is transmitted, via the olfactory tract, to the PC, which is located at the junction of the frontal and temporal lobes (Blazing and Franks, 2020). The PC is the largest recipient of neuronal input from the OB and therefore a major part of the primary olfactory cortex (including also other cortical areas like the amygdala and the entorhinal cortex). These areas work together to process not only the chemical properties of odorants, but also emotional and associative aspects of smell (Bao et al., 2016; Miao et al., 2021).

While olfactory processing in adults is well understood, the maturation of this system in newborns offers fascinating insights. Adam--Darque et al., (2018)) from the University of Geneva, examined with the functional MRI in the newborn the cortical responses to olfactory and trigeminal odorants. Trigeminal odorants are odorants associated with sensations of cooling (e.g., menthol) or irritation (e.g., ammonia), stimulate both olfactory and somatosensory pathways, involving pain and tactile processing. These stimuli provide a broader understanding of how the developing brain integrates multiple sensory inputs, which is crucial during the neonatal period when sensory systems are still maturing. They showed that the developing brain, only few days after birth, processes new artificial odorants in similar cortical areas than adults, including PC, entorhinal cortex, OFC, insula and ACC.

This early activation in neonatal brains suggests that the basic circuitry for olfactory processing is already established shortly after birth. However, the functional implications of these early responses may differ between neonates and adults due to the ongoing development of cognitive and associative processes.

### 2.3. Olfactory abilities in newborns: Early development and recognition

In newborns, the sense of smell enables the recognition of maternal odors and breast milk, helping to orient themselves in the environment and complete the physiological transition from intrauterine to extrauterine life (Schaal et al., 2020). Newborns identify and prefer maternal scents, such as the smell of their mother’s breast milk and amniotic fluid (Varendi et al., 1998; Marlier and Schaal, 2005; Contreras et al., 2013). This ability emerges within the first days of life and plays a vital role in the bonding process between mother and child. Maternal odors provide the newborn with a sense of safety and familiarity, helping to soothe and promote essential behaviors like feeding (Davidson et al., 2019).

Studies show that newborns, just days after birth, can distinguish their mother’s milk scent from that of other women (Badiee et al., 2013). This ability is linked to the development of the olfactory system during pregnancy, as the fetus is exposed to maternal odors through the amniotic fluid.

The discrimination of odorous molecules in amniotic fluid occurs after 30 weeks’ gestation (Sarnat, 1978). Fetuses exhibit differential responses to maternal diet (Mennella and Beauchamp., 1991; Mennella et al., 1995; Schaal et al., 2000; Wagner et al., 2019; Spahn et al., 2019) and can process olfactory stimuli present in the womb. This early exposure shapes their sensory preferences, as newborns continue to exhibit a preference for these familiar odors for months after birth (Tristão et al., 2021). Also, several studies indicate that, from birth, neonates’ olfactory abilities are strongly correlated with action systems. For instance, an odorant presentation elicits in the newborn different motor responses, such as the movement of the head towards the odorant or movements of the mouth and lips (Marlier and Schaal, 2005; Alberts and Ronca, 2012).

Maternal odors have been demonstrated also to exert a significant analgesic effect on newborns. When exposed to their mother’s breast milk or amniotic fluid, newborns tend to exhibit reduced pain responses during medical procedures, such as heel pricks or vaccinations (Wu et al., 2020; Lan et al., 2021). This exposure not only lowers physiological indicators of pain, such as heart rate and peripheral saturation of oxygen, but also significantly reduces crying time, suggesting a soothing and calming effect (Lin et al., 2022). Therefore, the ability of newborns to recognize and respond to maternal odors is crucial for their physiological adaptation and emotional well-being, facilitating bonding and reducing pain during early life challenges.

### 2.4. Olfactory memory and habituation

Olfactory perception also is linked to olfactory memory, which can be tested as a habituation response (Chaudhury et al., 2010). Olfactory memory is defined as recollection of odorants. In adults, habituation involves a process of central information integration with a gradual decrease in response to a repeated and known stimulus (Poellinger et al., 2001).

This phenomenon reflects the brain’s ability to filter out repetitive stimuli, allowing greater focus on novel or significant cues. Lipsitt and Rovee-Collier demonstrated that newborns could not only detect pure olfactory stimuli, as opposed to odorous trigeminal irritants, but also could habituate to them over repeated presentations (Lipsitt and Rovee-Collier, 2012). Moreover, Faas and coworkers conducted a study where, in a population of healthy term newborns, they administered different doses of amniotic fluid at three different timepoints. Each newborn was examined three times with a different dose each time, with the stimulus lasting for 15 seconds and then removed for 45 seconds. From the analysis of the response at different times, they showed that the maximum reactivity, in terms of general movements, was achieved during the first hour with a reduction at three hours and a return to baseline at 6 hours. Within the first hour the response was more significant during the first 15 seconds of stimulation with a reduction observed in the post stimulation intervals. The occurrence of this phenomenon in all tests supports the presence of a habituation process and allows a motor fatigue or receptor adaptation process (Faas et al., 2013).

### 2.5. Olfactory function impairment in other neurological diseases

Olfactory impairment is a potential early marker for the onset of neurodegenerative diseases, but the underlying mechanism is still poorly understood. The loss of smell is considered a clinical sign of early-stage disease and a marker of the disease’s progression and cognitive impairment (Rey et al., 2018; Liu et al., 2024). In Parkinson disease (PD), the olfactory impairment shows up in the early stages before the motor symptoms (Fullard et al., 2017) suggesting that the use of smell is a valuable tool for the characterization of the prodrome stages, in identifying early diagnostic strategies and in prediction of the outcomes (Guo et al., 2020).

Moreover, in Alzheimer (AD), PD and in stroke, several cortical and subcortical areas directly involved in olfactory function are damaged or show signs of atrophy, including the olfactory bulb, primary olfactory cortex (POC), hippocampus, OFC, amygdala, and olfactory tract (Iizuka et al., 2021).

Like in conditions such as AD, PD, and stroke, PA can impact both cortical and subcortical regions critical for olfactory perception. These regions include the hippocampus, OB, POC, amygdala, and OFC. The OB is particularly vulnerable to hypoxia, and damage to this structure can impair a newborn’s ability to process olfactory stimuli (Hernández-Soto et al., 2021).

Infants who survive hypoxic-ischemic episodes may face challenges in developing normal olfactory pathways, which can affect odor processing as well as functions like maternal bonding, feeding, and social behavior, potentially leading to long-term impacts on cognitive and social development.

## 3. Discussion and conclusions

Perinatal asphyxia is a significant cause of neonatal morbidity and mortality, leading to long-term neurodevelopmental deficits.

Currently, although there are some reliable tools that can predict long term consequences in infants with severe HIE, there remains a lack of comprehensive anatomical and functional tools to accurately assess the overall impact of HIE after PA on learning and other cognitive domains (Meys et al., 2022). Early tests, predictive of later abnormal impairment would be extremely important to identify infants at risk of sensory-motor, cognitive and learning abnormalities and crucial to the success of therapeutic interventions.

Olfactory dysfunction has emerged as a hallmark feature shared among several neurological conditions, including both neurodevelopmental and neurodegenerative disorders. While brain injury, following perinatal asphyxia, has been extensively studied for decades, especially in severe cases, its association with olfaction still lacks investigation. Olfactory sensing begins when odor molecules bind to diverse olfactory receptors on olfactory sensory neurons located within the olfactory epithelium in the nasal cavity. The activation of olfactory-related cortical regions so early in life suggests that the olfactory system is not only functional but also highly integrated with other sensory modalities. This integration may be critical for neonatal survival, as odors are involved in key processes such as mother-infant bonding, feeding behavior, and early environmental learning. The role of regions like the ACC and OFC in response to olfactory stimuli suggests that olfactory experiences may influence early cognitive and emotional development. Also, the ability to habituate to olfactory stimuli might serve as a marker of neurological health, potentially aiding in the identification of abnormalities in early brain development.

During early development, olfactory inputs are critical in synchronizing brain regions involved in olfactory and cognitive processing (Gretenkord et al., 2019; Kostka and Hanganu-Opatz, 2023). Reduced odor detection early in life, accompanied by anatomical and functional alterations in olfactory and higher-order cortical networks, has been reported in neurodevelopmental disorders as autistic spectrum disorder and attention deficit/hyperactivity disorder (Crow et al., 2020). Environmental factors play a significant role in shaping the development of the olfactory system and are implicated in neurodevelopmental disorders. PA has been demonstrated to be associated with extensive damage across multiple levels of the olfactory system, in experimental animal models. Notably, hypoxic ischemic injury during early development might impair the maturation of olfactory-hippocampal networks with significative effect on cognitive abilities later in life (Chen et al., 2023).

In neonatal intensive care units, olfactory habituation testing could serve as a valuable tool for the early detection of developmental disorders, particularly in newborns at high risk for neurodevelopmental impairments, such as those affected by PA and subsequent HIE of various degrees. Building on studies conducted with healthy newborns, odor stimuli could be delivered using disposable cotton swabs soaked in fragrant substances. For infants requiring non invasive ventilation or intubation, a custom-made olfactometer capable of automatically administering odorants could be employed (Adam-Darque et al., 2018; Arichi et al., 2013). Clinical follow-up incorporating cognitive, behavioral, sensory, and emotional assessments might further help predicting long-term neurodevelopmental outcomes in these infants.

In conclusion, HI damage to PC, OFC, amygdala and hippocampus could impair olfactory processing in infants, potentially impacting cognitive and social development. Understanding the interplay between olfactory system development and perinatal brain injury could pave the way for novel diagnostic and prognostic tools. Moreover, exploring the processes underlying olfactory habituation can also help in stratifying the risk groups for early (potential) and post-discharge follow-up interventions in infancy. Further research in both clinical and animal models is needed to explore the full potential of olfactory assessment in predicting outcomes following PA and HIE.

## Figures and Tables

**Table 1:** Hypoxic-ischemic encephalopathy and olfactory response in animal models.

Study	Intervention model	Animals	Study design/methods	Outcome
Drobyshevsky et al. (2006)	Intrauterine hypoxiaischemia	Rabbits	Manganese Enhanced MRI Olfactory Testing Histology and immunochemistry	Long-lasting injury to neuronal tracts of the olfactory system
Drobyshevsky et al. (2012)	Intrauterine hypoxiaischemia	Rabbits	MEMRI Administration of intranasal NO donors or a highly selective nNOS inhibitor. Gene expression of nNOS	Prenatal Hypoxia ischemia decreases NO-dependence of olfactory signal transduction.
Dubrovskaya et al. (2022)	Intrauterine acute normobaric hypoxia	Wistar rats	Odor preference test Food search productivity test Light Microscopy	Damage to multiple targets of the olfactory system, from the sensory neurons in the olfactory epithelium to cortical areas
Amakhin et al. (2022)	Intrauterine hypoxia	Wistar rats	Microscopy and Immunochemistry analysis Electrophysiology Dosed Electroshock	Neuronal loss in entorhinal cortex Hyperexcitability and lower seizure thresholds in limbic structures
Vasilev et al. (2021)	Intrauterine hypoxia	Wistar rats	mRNA analysis Odor preference test Food search test	Decreased levels of neprilysin (NEP) and number of dendritic spines in the hippocampus, entorhinal and parietal cortices
Shi et al. (2024)	Prenatal Hypoxia	Rabbits	Cognitive Recognition Tests	Conditioned newborn rabbits exhibit a preference for the face of the feeder but not for the laboratory coat.

## References

[R1] Adam-Darque A, Grouiller F, Vasung L, Ha-Vinh Leuchter R, Pollien P, Lazeyras F, Hüppi PS, 2018. fMRI-based Neuronal Response to New Odorants in the Newborn. Brain. Cereb. Cort 28 (8), 2901–2907. 10.1093/cercor/bhx167 29106509

[R2] Akter M, Kaneko N, Sawamoto K, 2021. Neurogenesis and neuronal migration in the postnatal ventricular-subventricular zones: similarities and dissimilarities between rodents and primates. Neurosci. Res 167, 64–69. 32553727 10.1016/j.neures.2020.06.001

[R3] Alberts JR, Ronca AE, 2012. The experience of being born: a natural context for learning to suckle. Int J. Pedia, 129328. 10.1155/2012/129328 PMC3463930 23056061

[R4] Amakhin DV, Soboleva EB, Postnikova TY, Tumanova NL, Dubrovskaya NM, Kalinina DS, Vasilev DS, Zaitsev AV, 2022. Maternal hypoxia increases the excitability of neurons in the entorhinal cortex and dorsal hippocampus of rat offspring. Front Neurosci. 16, 867120. 35495064 10.3389/fnins.2022.867120 PMC9042652

[R5] Aqrabawi AJ, Kim JC, 2018. Hippocampal projections to the anterior olfactory nucleus differentially convey spatiotemporal information during episodic odour memory. Nat. Commun 9, 2735. 30013078 10.1038/s41467-018-05131-6 PMC6048034

[R6] Arichi T, Gordon-Williams R, Allievi A, Groves AM, Burdet E, Edwards AD, 2013. Computer-controlled stimulation for functional magnetic resonance imaging studies of the neonatal olfactory system. Acta Paediatr. 102 (9), 868–875. 23789919 10.1111/apa.12327 PMC3795441

[R7] Azoulay R, Fallet-Bianco C, Garel C, Grabar S, Kalifa G, Adamsbaum C, 2006. MRI of the olfactory bulbs and sulci in human fetuses. Pedia Radio. 36 (2), 97–107. 10.1007/s00247-005-0030-0 16341529

[R8] Badiee Z, Asghari M, Mohammadizadeh M, 2013. The calming effect of maternal breast milk odor on premature infants. Pedia Neonatol. 54 (5), 322–325. 10.1016/j.pedneo.2013.04.004 23707040

[R9] Bao X, Raguet LL, Cole SM, Howard JD, Gottfried J, 2016. The role of piriform associative connections in odor categorization. eLife 5, e13732. 27130519 10.7554/eLife.13732 PMC4884078

[R10] Blazing RM, Franks KM, 2020. Odor coding in piriform cortex: mechanistic insights into distributed coding. Curr. Opin. Neurobiol 64, 96–102. 32422571 10.1016/j.conb.2020.03.001 PMC8782565

[R11] Bouiller JP, Dreyfus M, Mortamet G, Guillois B, Benoist G, 2016. Asphyxie perpartum à terme: facteurs de risque de survenue et conséquences à court terme. À propos de 82 cas. J. Gynecol., Obstet. Biol. Reprod 45 (6), 626–632. 10.1016/j.jgyn.2015.06.022 26321609

[R12] Branigan B, Tadi P Physiology, Olfactory. [Updated 2023 May 1]. In: StatPearls [Internet]. Treasure Island (FL): StatPearls Publishing; 2024 Jan-. Available from: https://www.ncbi.nlm.nih.gov/books/NBK542239/. 31194396

[R13] Bredt DS, Snyder SH, 1994. Nitric oxide: a physiologic messenger molecule. Ann. Rev. Biochem 63, 175–195. 7526779 10.1146/annurev.bi.63.070194.001135

[R14] Chaudhury D, Manella L, Arellanos A, Escanilla O, Cleland TA, Linster C, 2010. Olfactory bulb habituation to odor stimuli. Behavl Neurosci. 124 (4), 490–499. 10.1037/a0020293 PMC2919830 20695648

[R15] Chen YN, Kostka JK, Bitzenhofer SH, Hanganu-Opatz IL, 2023. Olfactory bulb activity shapes the development of entorhinal-hippocampal coupling and associated cognitive abilities. Curr. Biol. 33 (20), 4353–4366.e5. 37729915 10.1016/j.cub.2023.08.072 PMC10617757

[R16] Contreras CM, Gutiérrez-García AG, Mendoza-López R, Rodríguez-Landa JF, Bernal-Morales B, Díaz-Marte C, 2013. Amniotic fluid elicits appetitive responses in human newborns: fatty acids and appetitive responses. Dev. Psychobiol 55 3, 221–231. 22315200 10.1002/dev.21012

[R17] Cornet MC, Kuzniewicz M, Scheffler A, Forquer H, Hamilton E, Newman TB, Wu YW, 2023. Perinatal hypoxic-ischemic encephalopathy: incidence over time within a modern US birth cohort. Pediatr. Neurol 149, 145–150. 37883841 10.1016/j.pediatrneurol.2023.08.037 PMC10842130

[R18] Crow AJD, Janssen JM, Vickers KL, Parish-Morris J, Moberg PJ, Roalf DR, 2020. Olfactory dysfunction in neurodevelopmental disorders: a meta-analytic review of autism spectrum disorders. Atten. Deficit/Hyperact. Disord. Obsessive-Compuls. Disord. J. Autism Devel Dis 50 (8), 2685–2697. 10.1007/s10803-020-04376-9 PMC7369237 31960263

[R19] Davidson J, Ruthazer R, Maron JL, 2019. Optimal timing to utilize olfactory stimulation with maternal breast milk to improve oral feeding skills in the premature newborn. Breast Med 14 (4), 230–235. 10.1089/bfm.2018.0180 PMC10027347 30882237

[R20] Del Río R, Ochoa C, Alarcon A, Arnáez J, Blanco D, García-Alix A, 2016. Amplitude integrated electroencephalogram as a prognostic tool in neonates with hypoxic-ischemic encephalopathy: a systematic review. PloS One 11 (11), e0165744. 27802300 10.1371/journal.pone.0165744 PMC5089691

[R21] Drobyshevsky A, Robinson AM, Derrick M, Wyrwicz AM, Ji X, Englof I, Tan S, 2006. Sensory deficits and olfactory system injury detected by novel application of MEMRI in newborn rabbit after antenatal hypoxia-ischemia. NeuroImage 32 (3), 1106–1112. 16861007 10.1016/j.neuroimage.2006.06.002

[R22] Drobyshevsky A, Yu L, Yang Y, Khalid S, Luo K, Jiang R, Ji H, Derrick M, Kay L, Silverman RB, Tan S, 2012. Antenatal insults modify newborn olfactory function by nitric oxide produced from neuronal nitric oxide synthase. Exp. Neurol 237 (2), 427–434. 22836143 10.1016/j.expneurol.2012.07.006 PMC3443317

[R23] Dubie AG, Kokeb M, Mersha AT, Agegnehu CD, 2021. Prevalence and associated factors of perinatal asphyxia in newborns admitted to neonatal intensive care unit at the University of Gondar Comprehensive Specialized Hospital, Northwest Ethiopia, Ethiopia. BMC Pediatr. 21 (1), 525. 34837981 10.1186/s12887-021-03019-x PMC8626890

[R24] Dubrovskaya NM, Vasilev DS, Tumanova NL, Alekseeva OS, Nalivaeva NN, 2022. Prenatal hypoxia impairs olfactory function in postnatal ontogeny in rats. Neurosci. Behav. Physiol 52 (2), 262–270. 35317268 10.1007/s11055-022-01233-3 PMC8930458

[R25] Ekanayake A, Yang Q, Kanekar S, Ahmed B, McCaslin S, Kalra D, Eslinger P, Karunanayaka P, 2023. Monorhinal and Birhinal Odor Processing in Humans: an fMRI investigation. bioRxiv 08 (01), 551475. 10.1093/chemse/bjae038 PMC11582365 39387136

[R26] Faas AE, Resino CF, Moya PR, 2013. Neonatal responsiveness to the odor of amniotic fluid. Arch. Argent. Pedia 111 (2), 105–109. 10.5546/aap.2013.eng.105 23568065

[R27] Fullard ME, Morley JF, Duda JE, 2017. Olfactory dysfunction as an early biomarker in parkinson’s disease. Neurosci. Bull 33 (5), 515–525. 28831680 10.1007/s12264-017-0170-x PMC5636737

[R28] Gebregziabher GT, Hadgu FB, Abebe HT, 2020. Prevalence and associated factors of perinatal asphyxia in neonates admitted to ayder comprehensive specialized hospital, Northern Ethiopia: a cross-sectional study. Int J. Pedia 2020, 4367248. 10.1155/2020/4367248 PMC7042545 32110243

[R29] Goswami I, Guillot M, Tam EWY, 2020. Predictors of long-term neurodevelopmental outcome of hypoxic-ischemic encephalopathy treated with therapeutic hypothermia. Semin Neurol. 40 (3), 322–334. 32079030 10.1055/s-0040-1702939

[R30] Gretenkord S, Kostka JK, Hartung H, Watznauer K, Fleck D, Minier-Toribio A, Spehr M, Hanganu-Opatz IL, 2019. Coordinated electrical activity in the olfactory bulb gates the oscillatory entrainment of entorhinal networks in neonatal mice. PLoS Biol. 17 (1), e2006994. 30703080 10.1371/journal.pbio.2006994 PMC6354964

[R31] Guo P, Wang RD, Lian TH, Ding DY, Zhang YN, Zhang WJ, Li DN, Li LX, Li JH, Guan HY, Yu SY, Liu L, Hu Y, Zuo LJ, Yu QJ, Wang XM, Zhang W, 2020. Olfactory dysfunction and its association with neuropathologic proteins in cerebrospinal fluid from patients with parkinson disease. Front Aging Neurosci. 12, 594324. 33362530 10.3389/fnagi.2020.594324 PMC7759606

[R32] Hernández-Soto R, Villasana-Salazar B, Pinedo-Vargas L, Penã-Ortega F, 2021. Chronic intermittent hypoxia alters main olfactory bulb activity and olfaction. Exp. Neurol 340, 113653. 33607078 10.1016/j.expneurol.2021.113653

[R33] Iizuka N, Masaoka Y, Kubota S, Sugiyama H, Yoshida M, Yoshikawa A, Koiwa N, Honma M, Watanabe K, Kamijo S, Kamimura S, Ida M, Ono K, Izumizaki M, 2021. Entorhinal cortex and parahippocampus volume reductions impact olfactory decline in aged subjects. Brain Behav. 11 (5), e02115. 33769719 10.1002/brb3.2115 PMC8119819

[R34] Kebaya LMN, Kapoor B, Mayorga PC, Meyerink P, Foglton K, Altamimi T, Nichols ES, de Ribaupierre S, Bhattacharya S, Tristao L, Jurkiewicz MT, Duerden EG, 2023. Subcortical brain volumes in neonatal hypoxic-ischemic encephalopathy. Pedia Res 94 (5), 1797–1803. 10.1038/s41390-023-02695-y 37353661

[R35] Kostka JK, Hanganu-Opatz IL, 2023. Olfactory-driven beta band entrainment of limbic circuitry during neonatal development. J. Phys 601 (16), 3605–3630. 10.1113/JP284401 37434507

[R36] Lan HY, Yang L, Lin CH, Hsieh KH, Chang YC, Yin T, 2021. Breastmilk as a multisensory intervention for relieving pain during newborn screening procedures: a randomized control trial. Int J. Environ. Res Public Health 18 (24), 13023. 34948633 10.3390/ijerph182413023 PMC8701293

[R37] Lee BL, Glass HC, 2021. Cognitive outcomes in late childhood and adolescence of neonatal hypoxic-ischemic encephalopathy. Clin. Exp. Pedia 64 (12), 608–618. 10.3345/cep.2021.00164 PMC8650814 34044480

[R38] Lin CH, Liaw JJ, Chen YT, Yin T, Yang L, Lan HY, 2022. Efficacy of breast milk olfactory and gustatory interventions on neonates’ biobehavioral responses to pain during heel prick procedures. Int J. Environ. Res Public Health 19 (3), 1240. 35162263 10.3390/ijerph19031240 PMC8834920

[R39] Lipchock SV, Reed DR, Mennella JA, 2011. The gustatory and olfactory systems during infancy: implications for development of feeding behaviors in the high-risk neonate. Clin. Perinatol 38 (4), 627–641. 22107894 10.1016/j.clp.2011.08.008 PMC3223371

[R40] Lipsitt LP, Rovee-Collier C, 2012. In: Zucco In.G.M., Herz RS, Schaal B (Eds.), The psychophysics of olfaction in the human newborn: Habituation and cross-adaptation. Olfactory cognition, pp. 221–235.

[R41] Liu D, Lu J, Wei L, Yao M, Yang H, Lv P, Wang H, Zhu Y, Zhu Z, Zhang X, Chen J, Yang QX, Zhang B, 2024. Olfactory deficit: a potential functional marker across the Alzheimer’s disease continuum. Front Neurosci. 18, 1309482. 38435057 10.3389/fnins.2024.1309482 PMC10907997

[R42] Logan DW, Brunet LJ, Webb WR, Cutforth T, Ngai J, Stowers L, 2012. Learned recognition of maternal signature odors mediates the first suckling episode in mice. Curr. Biol 22 (21), 1998–2007. 23041191 10.1016/j.cub.2012.08.041 PMC3494771

[R43] Marlier L, Schaal B, 2005. Human newborns prefer human milk: conspecific milk odor is attractive without postnatal exposure. Child Dev. 76 (1), 155–168. 15693764 10.1111/j.1467-8624.2005.00836.x

[R44] Mennella JA, Beauchamp GK, 1991. Maternal diet alters the sensory qualities of human milk and the nursling’s behavior. Pediatrics 88 (4), 737–744. 1896276

[R45] Mennella JA, Johnson A, Beauchamp GK, 1995. Garlic ingestion by pregnant women alters the odor of amniotic fluid. Chem. Senses 20 (2), 207–209. 7583013 10.1093/chemse/20.2.207

[R46] Meys KME, de Vries LS, Groenendaal F, Vann SD, Lequin MH, 2022. The mammillary bodies: a review of causes of injury in infants and children. Ajnr. Am. J. Neuroradiol 43 (6), 802–812. 35487586 10.3174/ajnr.A7463 PMC9172959

[R47] Miao X, Paez AG, Rajan S, Cao D, Liu D, Pantelyat AY, Rosenthal LI, van Zijl PCM, Bassett SS, Yousem DM, Kamath V, Hua J, 2021. Functional activities detected in the olfactory bulb and associated olfactory regions in the human brain using T2-prepared BOLD functional MRI at 7T. Front Neurosci. 15, 723441. 34588949 10.3389/fnins.2021.723441 PMC8476065

[R48] Mitra S, Bale G, Highton D, Gunny R, Uria-Avellanal C, Bainbridge A, Sokolska M, Price D, Huertas-Ceballos A, Kendall GS, Meek J, Tachtsidis I, Robertson NJ, 2019. Pressure passivity of cerebral mitochondrial metabolism is associated with poor outcome following perinatal hypoxic ischemic brain injury. J. Cerebr Blood Flow. Metab 39 (1), 118–130. 10.1177/0271678X17733639 PMC6311664 28949271

[R49] Mota-Rojas D, Villanueva-García D, Solimano A, Muns R, Ibarra-Ríos D, Mota-Reyes A, 2022. Pathophysiology of perinatal asphyxia in humans and animal models. Biomedicines 10 (2), 347. 35203556 10.3390/biomedicines10020347 PMC8961792

[R50] Negro S, Benders MJNL, Tataranno ML, Coviello C, de Vries LS, van Bel F, Groenendaal F, Longini M, Proietti F, Belvisi E, Buonocore G, Perrone S, 2018. Early prediction of hypoxic-ischemic brain injury by a new panel of biomarkers in a population of term newborns. Oxid. Med Cell Longev 2018, 7608108. 30050660 10.1155/2018/7608108 PMC6046131

[R51] Nguefack S, Tebon FL, Tague DAK, Tetinou FD, Tetinou NA, Nguefack F, Mah E, Chiabi A, 2022. Long term outcome of neonates with hypoxic ischemic encephalopathy. Pedia Oncall J. 19, 10–14.

[R52] Nocera S, Simon A, Fiquet O, Chen Y, Gascuel J, Datiche F, Schneider N, Epelbaum J, Viollet C, 2019. Somatostatin serves a modulatory role in the mouse olfactory bulb: neuroanatomical and behavioral evidence. Front Behav. Neurosci 13, 61. 31024270 10.3389/fnbeh.2019.00061 PMC6465642

[R53] Poellinger A, Thomas R, Lio P, Lee A, Makris N, Rosen BR, Kwong KK, 2001. Activation and habituation in olfaction–an fMRI study. NeuroImage 13 (4), 547–560. 11305885 10.1006/nimg.2000.0713

[R54] Purves D, Augustine GJ, Fitzpatrick D, , editors. Neuroscience. 2nd edition. Sunderland (MA); (2001). Sinauer Associates. The Olfactory Epithelium and Olfactory Receptor Neurons. Available from: ⟨https://www.ncbi.nlm.nih.gov/boo ks/NBK10896/⟩.

[R55] Rey NL, Wesson DW, Brundin P, 2018. The olfactory bulb as the entry site for prionlike propagation in neurodegenerative diseases. Neurobiol. Dis 109 (Pt B), 226–248. 28011307 10.1016/j.nbd.2016.12.013 PMC5972535

[R56] Rilling JK, Young LJ, 2014. The biology of mammalian parenting and its effect on offspring social development. Science 345, 771–776. 25124431 10.1126/science.1252723 PMC4306567

[R57] Roskams AJ, Bredt DS, Dawson TM, Ronnett GV, 1994. Nitric oxide mediates the formation of synaptic connections in developing and regenerating olfactory receptor neurons. Neuron 13 (2), 289–299. 7520251 10.1016/0896-6273(94)90347-6

[R58] Sarnat HB, 1978. Olfactory reflexes in the newborn infant. J. Ped 92 (4), 624–626. 10.1016/s0022-3476(78)80307-2 633025

[R59] Sarnat HB, Yu W, 2016. Maturation and dysgenesis of the human olfactory bulb. Brain Pathol. (Zur., Switz. ) 26 (3), 301–318. 10.1111/bpa.12275 PMC8028954 26096058

[R60] Sarnat HB, Flores-Sarnat L, Wei XC, 2017. Olfactory development, part 1: function, from fetal perception to adult wine-tasting. J. Child Neurol 32 (6), 566–578. 28424010 10.1177/0883073817690867

[R61] Schaal B, Marlier L, Soussignan R, 2000. Human foetuses learn odours from their pregnant mother’s diet. Chem. Senses 25 (6), 729–737. 11114151 10.1093/chemse/25.6.729

[R62] Schaal B, Saxton TK, Loos H, Soussignan R, Durand K,2020. Olfaction scaffolds the developing human from neonate to adolescent and beyond. Philos. Trans. R. Soc. Lond. B Biol. Sci 375 (1800), 20190261. 32306879 10.1098/rstb.2019.0261 PMC7209940

[R63] Sharma A, Kumar R, Aier I, Semwal R, Tyagi P, Varadwaj P, 2019. Sense of smell: structural, functional, mechanistic advancements and challenges in human olfactory research. Curr. Neuropharmacol 17 (9), 891–911. 30520376 10.2174/1570159X17666181206095626 PMC7052838

[R64] Shi Z, Luo K, Deol S, Tan S, 2022a. A systematic review of noninflammatory cerebrospinal fluid biomarkers for clinical outcome in neonates with perinatal hypoxic brain injury that could be biologically significant. J. Neurosci. Res 100 (12), 2154–2173. 33543500 10.1002/jnr.24801 PMC9249405

[R65] Shi Z, Luo K, Jani S, February M, Fernandes N, Venkatesh N, Sharif N, Tan S, 2022b. Mimicking partial to total placental insufficiency in a rabbit model of cerebral palsy. J. Neurosci. Res 100 (12), 2138–2153. 34173261 10.1002/jnr.24901 PMC8709884

[R66] Shi Z, Sharif N, Luo K, Tan S, 2024. Development of a new scoring system in higher animals for testing cognitive function in the newborn period: effect of prenatal hypoxia-ischemia. Dev. Neurosci 10.1159/000538607. PMC11436483 38547848

[R67] Smith TD, Bhatnagar KP, 2019. Anatomy of the olfactory system. Handb. Clin. Neurol 164, 17–28. 31604545 10.1016/B978-0-444-63855-7.00002-2

[R68] Spahn JM, Callahan EH, Spill MK, Wong YP, Benjamin-Neelon SE, Birch L, Black MM, Cook JT, Faith MS, Mennella JA, Casavale KO, 2019. Influence of maternal diet on flavor transfer to amniotic fluid and breast milk and children’s responses: a systematic review. Am. J. Clin. Nutr 109 (_7), 1003S–1026S. 30982867 10.1093/ajcn/nqy240

[R69] Touhara K, Vosshall LB, 2009. Sensing odorants and pheromones with chemosensory receptors. Annu Rev. Physiol 71, 307–332. 19575682 10.1146/annurev.physiol.010908.163209

[R70] Tristão RM, Lauand L, Costa KSF, Brant LA, Fernandes GM, Costa KN, Spilski J, Lachmann T, 2021. Olfactory sensory and perceptual evaluation in newborn infants: a systematic review. Dev. Psychobiol 63 (7), e22201. 34674234 10.1002/dev.22201

[R71] Tufo C, Poopalasundaram S, Dorrego-Rivas A, Ford MC, Graham A, Grubb MS, 2022. Development of the mammalian main olfactory bulb. Development 149 (3). 10.1242/dev.200210 PMC8918810 35147186

[R72] Varendi H, Christensson K, Porter RH, Winberg J, 1998. Soothing effect of amniotic fluid smell in newborn infants. Early Hum. Dev 51 (1), 47–55. 9570031 10.1016/s0378-3782(97)00082-0

[R73] Vasilev DS, Dubrovskaya NM, Zhuravin IA, Nalivaeva NN, 2021. Developmental profile of brain neprilysin expression correlates with olfactory behaviour of rats. J. Mol. Neurosci: MN 71 (9), 1772–1785. 33433852 10.1007/s12031-020-01786-3

[R74] Wagner S, Issanchou S, Chabanet C, Lange C, Schaal B, Monnery-Patris S, 2019. Weanling infants prefer the odors of green vegetables, cheese, and fish when their mothers consumed these foods during pregnancy and/or lactation. Chem. Senses 44 (4), 257–265. 30859182 10.1093/chemse/bjz011

[R75] Wilson CH, Christensen TA, Nighorn AJ, 2007. Inhibition of nitric oxide and soluble guanylyl cyclase signaling affects olfactory neuron activity in the moth, Manduca sexta. J. Comp. Physiol 193 (7), 715–728. 17551736 10.1007/s00359-007-0227-9 PMC2629079

[R76] Wilson DA, Sullivan RM, 2011. Cortical processing of odor objects. Neuron 72 (4), 506–519. 22099455 10.1016/j.neuron.2011.10.027 PMC3223720

[R77] Wu HP, Yin T, Hsieh KH, Lan HY, Feng RC, Chang YC, Liaw JJ, 2020. Integration of different sensory interventions from mother’s breast milk for preterm infant pain during peripheral venipuncture procedures: a prospective randomized controlled trial. J. Nurs. Sch 52 (1), 75–84. 10.1111/jnu.12530 31762179

[R78] Zhao Y, Bhutani S, Kahnt T, 2023. Appetite-regulating hormones modulate odor perception and odor-evoked activity in hypothalamus and olfactory cortices. Chem. Senses 48. 10.1093/chemse/bjad039 PMC10590159 37796827

